# SBMLSimulator: A Java Tool for Model Simulation and Parameter Estimation in Systems Biology

**DOI:** 10.3390/computation2040246

**Published:** 2014-12-18

**Authors:** Alexander Dörr, Roland Keller, Andreas Zell, Andreas Dräger

**Affiliations:** 1 Center for Bioinformatics Tuebingen (ZBIT), University of Tuebingen, Sand 1, 72076 Tübingen, Germany; 2 Systems Biology Research Group, University of California, San Diego, 9500 Gilman Drive, La Jolla, CA 92093, USA

**Keywords:** Systems Biology Markup Language (SBML), ordinary differential equation (ODE) modeling, simulation, parameter estimation

## Abstract

The identification of suitable model parameters for biochemical reactions has been recognized as a quite difficult endeavor. Parameter values from literature or experiments can often not directly be combined in complex reaction systems. Nature-inspired optimization techniques can find appropriate sets of parameters that calibrate a model to experimentally obtained time series data. We present SBMLsimulator, a tool that combines the Systems Biology Simulation Core Library for dynamic simulation of biochemical models with the heuristic optimization framework EvA2. SBMLsimulator provides an intuitive graphical user interface with various options as well as a fully-featured command-line interface for large-scale and script-based model simulation and calibration. In a parameter estimation study based on a published model and artificial data we demonstrate the capability of SBMLsimulator to identify parameters. SBMLsimulator is useful for both, the interactive simulation and exploration of the parameter space and for the large-scale model calibration and estimation of uncertain parameter values.

## 1. Introduction

The dynamic simulation of quantitative biological models belongs to the key aspects of research in systems biology [1]. Biological network models are typically encoded in Extensible Markup Language (XML)-based description formats [2], such as the Systems Biology Markup Language (SBML) [3]. In the most common scenario, such a model can be interpreted in terms of an ordinary differential equation (ODE) system with additional constructs, such as discrete events, delays, or algebraic rules [4,5]. A graphical display of the resulting curves can greatly facilitate the analysis of the system. The calculation of these dynamics requires the initial values of each model component to be known, including the concentration of reactive species as well as parameters, such as Michaelis constants. However, in many cases some of these values are missing or at least uncertain [6,7]. This makes their estimation or adjustment with respect to given experimental data necessary [8] (e.g., available in Gene Expression Omnibus [9] or KiMoSys databases [10]). To this end, heuristic optimization routines can be applied to fit the model to the experimental data. Nature-inspired optimization methods are known to handle even highly nonlinear optimization problems [11]. The basic idea of these methods is that many natural processes can be seen as optimization tasks, for instance the evolution of a population or the flight of a swarm of birds. In several applications, the Java^™^ framework EvA2 [12] has been shown to be promising for the optimization of biological systems [11,13–16].

Many systems biology simulation and optimization frameworks are available, e.g., AMIGO [17], SBToolbox2 [18], SBML-PET [19], COPASI [20], or Potters-Wheel [21]. The focus of SBMLsimulator is to provide the scientific community with an easily usable and flexible parameter estimation tool that understands and supports all aspects of the modeling format SBML through all of its levels and versions. Programs that do not completely cover the full standard, might have run-time advantages under certain conditions, but cannot guarantee that all models can be solved. Important features of SBMLsimulator include that it (a) is a free platform-independent open-source solution (b) does not depend on any commercial software (c) fully supports all specifications of SBML (d) comes with an easily usable program layout.

The SBMLsimulator project unifies two powerful libraries in one tool with a common user interface. Consequently, SBMLsimulator benefits from all the optimization algorithms provided by EvA2 [12] and as well as from all the modeling languages and ODE solvers that the Systems Biology Simulation Core Library (SBSCL) makes available. This modular program design opens the door to independently extend and exchange both libraries. SBMLsimulator hence evolves with the improvements of either one of its underlying libraries. With little effort, SBMLsimulator can then be modified to take advantage on all enhancements. The SBSCL already supports all elements of SBML and comprises several ODE solvers including a solver for stiff ODE systems (for a comparison of SBSCL to related simulation frameworks see [5] and the up-to-date comparisons of the SBML standard compliance [22]). EvA2 provides a plethora of nature-inspired heuristic optimization algorithms, such as evolution strategies [23], genetic algorithm [24], differential evolution [25], and particle swarm optimization [26], and many more. Teams of core developers maintain both open-source libraries SBSCL and EvA2 that can also be easily extended by the scientific community.

## 2. Implementation

The simulation of a model is done with the SBSCL [5] and JSBML [27] is used as internal data structure for model representation. The SBSCL outputs the simulation results in a specific format, which enables SBMLsimulator to plot the time course of the model’s species.

The basis of all those routines is a target function for model calibration, the so-called *fitness* function. Given a set of parameter values and experimental data, this function returns a value to EvA2 that reflects the quality of the current model configuration. That is, how precise the given set of parameters can reproduce the experimental data. For model calibration, the fitness is a distance function between simulation output and experimental data. Both, simulation and distance, are computed by the SBSCL. EvA2 optimizes the parameters of the model with respect to the fitness function. Hence, EvA2 tries to find a parameter set that accounts for the smallest possible deviation of simulation results and the given experimental data set. Before a parameter estimation can be performed, optimization targets and search intervals need to be specified by the user (see description in Results section).

The estimation with EvA2 can take several hours or days, as for each fitness evaluation a simulation has to be conducted. Hence, the time of one simulation run greatly influences the time needed for parameter estimation. Such parameter estimations will often be conducted in the command-line mode, which is an alternative to the graphical user interface (GUI) that SBMLsimulator launches by default. However, in the GUI mode the simulation results of the current parameter combination producing the best fitness are always plotted in SBMLsimulator. This enables the user to investigate intermediate results.

## 3. Results and Discussion

SBMLsimulator is straightforward to use and runs on every platform where a Java Virtual Machine is available. Its graphical user interface provides an intuitive way for displaying the curves of model entities chosen by the user. In addition, it comprises a graphical and command-line user interface that both provide a connection to EvA2. The program estimates all uncertain quantities with respect to given time-series of metabolite or gene expression values.

### 3.1. Graphical User Interface

**Figure 1.**
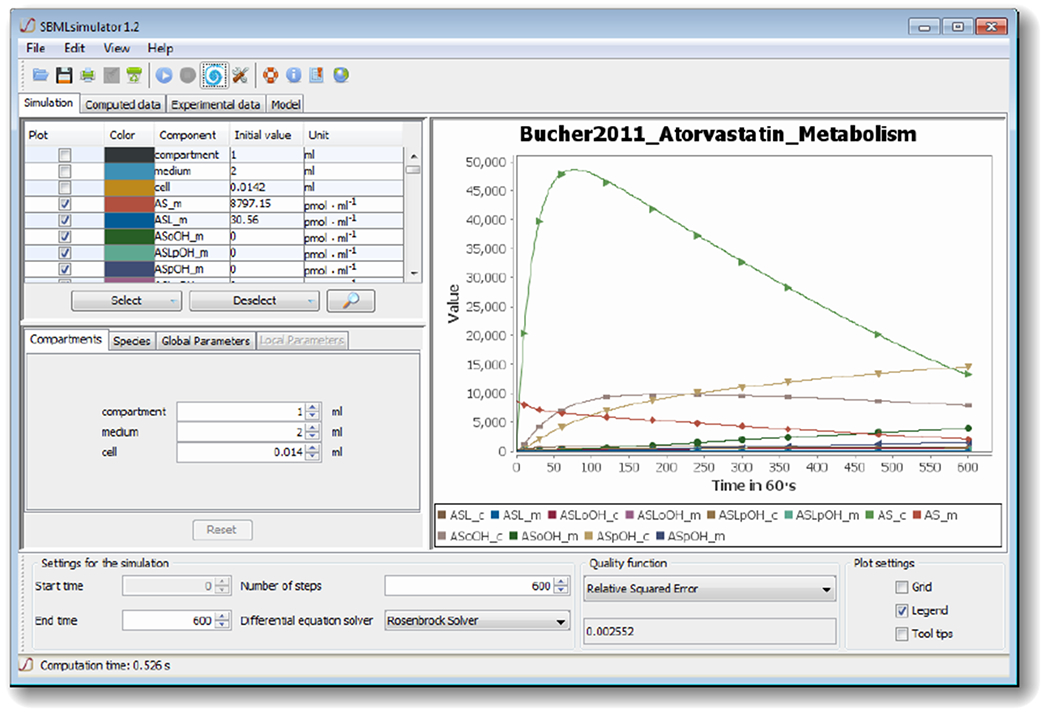
The graphical user interface (GUI) of The Systems Biology Markup Language (SBML)simulator. The figure shows the main window of SBMLsimulator after importing the model by Bucher *et al.* [28]. SBMLsimulator enables the user to modify initial quantities (middle left part of window) and to choose the quantities for plotting (upper left). Furthermore, at the bottom of the window the user can specify settings for simulation, such as the integration routine, the simulation start and end time, the simulation step size, and the quality function for comparing the simulated data to experimental data. The simulation can be started by clicking on the simulation button. The right part shows an intermediate solution, whereby the original values are depicted by shapes and the simulated values dependent on the current set of parameters are shown as curves. In the given state, the parameter optimization already found a set of parameters that fit the predefined values with a small error.

The graphical user interface (see Figure 1) comprises several separated sub-windows for the presentation of simulation results as well as for settings that can be modified by the user: at the lower part of the GUI, SBMLsimulator enables the user to choose the numerical solver and to set the start and end point as well as the step size of the simulation. For some solvers an error tolerance can also be specified under Edit/Preferences. The user also has the choice between different quality functions for calculating the distance between simulated and experimental data. The upper left part of the window allows the user to select, which model quantities should be plotted. Furthermore, the compartment, parameter and initial values of metabolites can be modified in the middle left part. Finally, the simulation of the model with the chosen settings can be conducted by one of the GUI’s control elements.

A dedicated dialog assists with the import of experimental data. SBMLsimulator suggests the most intuitive mapping of columns in the experimental data to model quantities. The user can then confirm this suggestion or adapt the mapping accordingly. The given experimental data are plotted together with the simulation results (as shown in Figure 1) in the user interface in order to facilitate the comparison with the simulation results of the model. Furthermore, after model simulation, the distance of the simulated data to the loaded experimental data is computed and displayed at the bottom of the SBMLsimulator window.

Besides the main window (Simulation), which shows the simulation results and enables the modification of simulation settings, there are also windows for displaying the simulation data (Computed data) and the imported experimental data (Experimental data) in table format. Furthermore, the user has the possibility to investigate the structure of the model in another window (Model).

Besides a comparison with simulated values, the import of experimental data facilitates a parameter estimation with respect to that data is possible. The user needs to specify the parameters as well as their ranges in a dedicated window. An alternative provided to set the parameters in this window, is to import a text file listing the parameters to estimate and their ranges. After the user has chosen the parameters and their limits, the user can select the type of evolutionary algorithm and change specific settings of the desired routine in EvA2. Eva2 lets the user choose between a large number of different optimization routines including hill climbing [29], simulated annealing [30], evolution strategies [23,31] with support for covariance matrix adaptation [32], genetic algorithm [24], differential evolution [25,33], and particle swarm optimization [26,34,35] as well as multi-modal approaches [15].

### 3.2. Parameter Estimation Study

In order to assess the capabilities of SBMLsimulator, we estimated the parameters of a model by Bucher *et al.* [28] explaining the biotransformation of atorvastatin (model BIOMD0000000328 of BioModels Database [36]). We first obtained an artificial data set by simulating the model with the respective start and end time described in the publication. We saved the simulated data and extracted time points similar to those used in the publication. This data set was then read in by SBMLsimulator, the parameters were deleted from the model, and optimization with EvA2 was launched with respect to the artificial data set using differential evolution [25]. The same parameters as in the publication were estimated with the intervals given in Table 1.

Here initial minimum/maximum stands for the interval, in which the parameters are randomly initiated, and minimum/maximum for the interval during the estimation procedure. We chose the same intervals for initiation and parameter estimation. The numerical integration was conducted with a Rosenbrock solver [37] (absolute error tolerance: 10^−12^, relative error tolerance: 10^−6^), as this routine is suitable for solving stiff differential equation systems. As fitness function we used the relative squared error, which has been suggested by, e.g., Dräger *et al.* [11,38].

**Figure 2.**
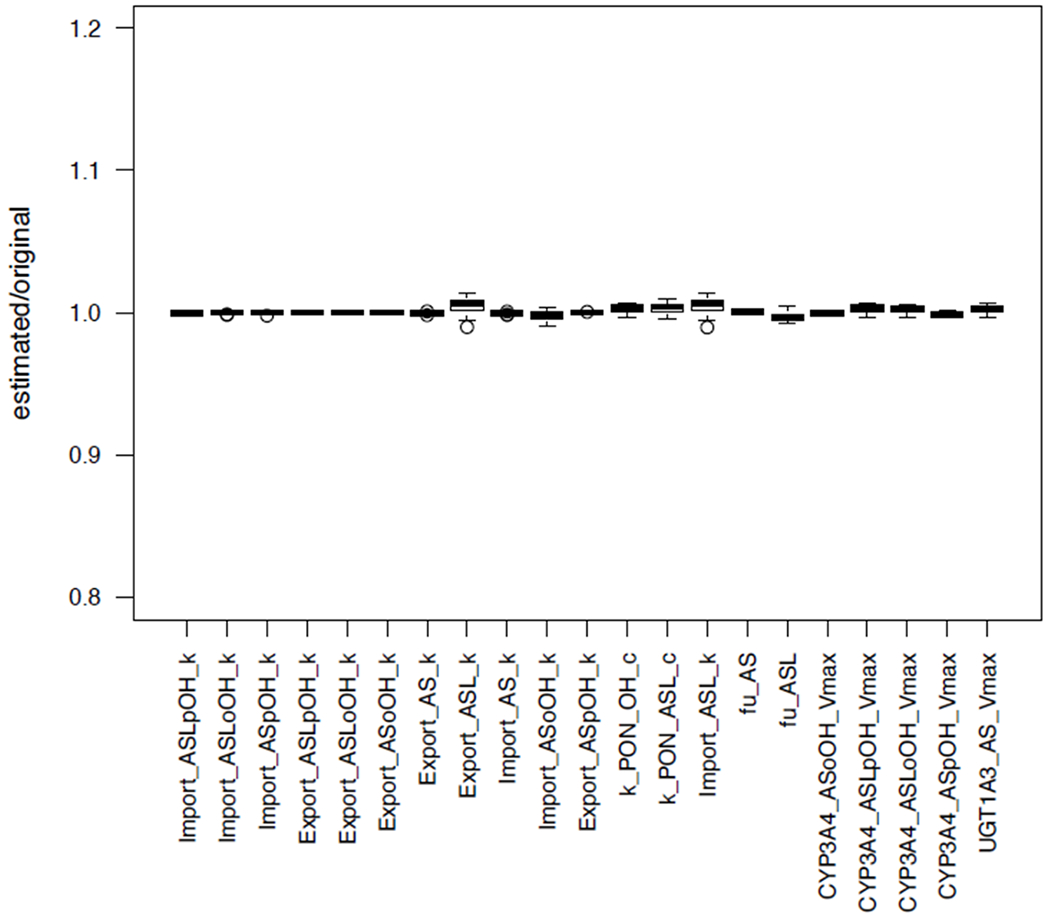
Distribution of parameters estimated with SBMLsimulator. One hundred parameter estimations with SBMLsimulator for the model by Bucher *et al.* [28] were run on a computer cluster. The distribution of the 50 estimations with the best fitness values is shown here. For each parameter the estimated values were divided by the original parameter value prior to plotting. It is obvious from the plot that all parameters were estimated closely around their original values. The figure has been created with R software package [40].

During an optimization, SBMLsimulator plots the best simulation curves together with the respective data each time it has finished processing a new generation of parameter combinations. Figure 1 shows such an intermediate result for the artificial data set. Hence, the user can nicely see how the distance between the simulated time course and data set drastically decreases over time. For such large-scale parameter estimations it is most suitable to run SBMLsimulator in command-line mode, because it can take a long time (see Section 5) and this task can then be performed on a computer cluster. Running parameter estimations on a computer cluster is also recommended, because estimations are usually conducted multiple times. This is done because an optimization run can get stuck in a local optimum of the fitness function. Another reason for repeating a parameter estimation multiple times is that this is a straightforward way to assess whether the estimated quantities are identifiable [39]. In order to test the identifiability of the parameters with SBMLsimulator, we estimated the model quantities of the atorvastatin biotransformation model 100 times on a computer cluster. Afterwards those 50% of the parameter estimates with the best fits were extracted and the distributions of the estimated quantity values were plotted (see Figure 2). It can be seen that the estimated parameter values had a low standard deviation, which means that SBMLsimulator could reliably identify these parameters. A very high standard deviation of a parameter would in contrast to that have suggested that this parameter could not be identified.

The results of this experiment show that our linkage of parameter estimation and simulation works well.

## 4. Conclusions

With SBMLsimulator we provide a freely usable platform-independent tool for simulation and parameter estimation of biochemical models, which fully supports all SBML elements. It combines two powerful toolboxes under one graphical interface: SBSCL for simulation and the nature-inspired optimization framework EvA2 for estimation of uncertain parameter values. SBMLsimulator is useful for both, the interactive simulation and exploration of the parameter space and for the large-scale model calibration. With a parameter estimation study based on a published model we have demonstrated its capability to identify model parameters with respect to experimental data. SBMLsimulator allows users to easily run models and to calibrate them to their experiments, to learn about simulation, or to gain new insight into the model’s behavior by modifying individual parameters or model components. All this is facilitated by the platform independence of SBMLsimulator. Since the SBSCL is also an open-source library, the user can even modify how different elements of a model are interpreted and learn from deviating simulation results. For large-scale simulation experiments, the use of native libraries can offer an advantage with respect to calculation speed. However, with a comprehensive command-line interface SBMLsimulator can also easily be deployed on a computer cluster to perform several simulations in parallel. A comprehensive users’ guide is available on the project’s homepage, which describes all program features and their use in detail.

In future versions of SBMLsimulator we are planning to include the possibility that the parameter search is conducted in log-scale. As the parameters are often of different magnitudes, this log-scale can be of advantage [16]. In order to satisfy the growing interest in other modeling languages like the Cell Markup Language (CellML) [41], SBMLsimulator should also be able to simulate models given in a different format. To this end, the SBSCL needs to be extended and after some minor modifications SBMLsimulator will gain the capability to simulate models in different formats, such as the simulation of models given in CellML similar to CellMLSimulator [42].

## 5. Availability and Requirements

The current version of SBMLsimulator is available at the project’s homepage as a runnable Java^™^ archive file (JAR) together with a documentation of the program.

**Project name:** SBMLsimulator

**Project homepage:** http://www.cogsys.cs.uni-tuebingen.de/software/SBMLsimulator/

**Contact:** sbmlsimulator@googlegroups.com

**Operating systems:** Platform independent, i.e., for all systems for which a Java^™^ Virtual Machine (JVM) is available.

**Programming language:** Java^™^

**Other requirements:** Java^™^ Runtime Environment (JRE) 1.6 or above

**License:** GNU Lesser General Public License (LGPL) version 3

## Figures and Tables

**Table 1.** Estimated parameters with units, their initial intervals and their intervals throughout parameter estimation for the model by Bucher *et al.* [28].

Parameter	Unit	Minimum Initial Value	Maximum Initial Value	Minimum Value	Maximum Value
Import_ASLpOH_k	mL · min^−1^	10^−6^	0.1	10^−6^	0.1
Import_ASLoOH_k	mL · min^−1^	10^−6^	0.1	10^−6^	0.1
Import_ASpOH_k	mL · min^−1^	10^−6^	0.1	10^−6^	0.1
Export_ASLpOH_k	mL · min^−1^	10^−6^	0.1	10^−6^	0.1
Export_ASLoOH_k	mL · min^−1^	10^−6^	0.1	10^−6^	0.1
Export_ASoOH_k	mL · min^−1^	10^−6^	0.1	10^−6^	0.1
Export_AS_k	mL · min^−1^	10^−6^	0.1	10^−6^	0.1
Export_ASL_k	mL · min^−1^	10^−6^	0.1	10^−6^	0.1
Import_AS_k	mL · min^−1^	10^−6^	0.1	10^−6^	0.1
Import_ASoOH_k	mL · min^−1^	10^−6^	0.1	10^−6^	0.1
Export_ASpOH_k	mL · min^−1^	10^−6^	0.1	10^−6^	0.1
k_PON_OH_c	mL · min^−1^	10^−6^	0.1	10^−6^	0.1
k_PON_ASL_c	mL · min^−1^	10^−6^	0.1	10^−6^	0.1
Import_ASL_k	mL · min^−1^	10^−6^	1	10^−6^	1
fu_AS	dimensionless	10^−6^	1	10^−6^	1
fu_ASL	dimensionless	10^−6^	1	10^−6^	1
CYP3A4_ASoOH_Vmax	pmol · min^−1^	10^−6^	100	10^−6^	100
CYP3A4_ASLpOH_Vmax	pmol · min^−1^	10^−6^	100	10^−6^	100
CYP3A4_ASLoOH_Vmax	pmol · min^−1^	10^−6^	100	10^−6^	100
CYP3A4_ASpOH_Vmax	pmol · min^−1^	10^−6^	100	10^−6^	100
UGT1A3_AS_Vmax	pmol · min^−1^	10^−6^	100	10^−6^	100

## Abbreviations/Nomenclature

AMIGO: Advanced Model Identification in systems biology using Global Optimization
COPASI: COmplex PAthway SImulator
CellML: Cell Markup Language
GUI: graphical user interface
JAR: Java^™^ archive file
JRE: Java^™^ Runtime Environment
JSBML: Java^™^ SBML
JVM: Java^™^ Virtual Machine
LGPL: GNU Lesser General Public License
ODE: ordinary differential equation
SBML: Systems Biology Markup Language
SBML-PET: SBML Parameter Estimation Tool
SBSCL: Systems Biology Simulation Core Library
XML: Extensible Markup Language
